# Effects of GPR110 expression on neurobehavioral outcomes in mice

**DOI:** 10.3389/fnins.2026.1774433

**Published:** 2026-03-04

**Authors:** Mariam Melkumyan, Bill X. Huang, Joel Toro, Andrew Kesner, Hee-Yong Kim

**Affiliations:** 1 Laboratory of Molecular Signaling, National Institute on Alcohol Abuse and Alcoholism, National Institutes of Health, Rockville, MD, United States; 2 Unit on Motivation and Arousal, National Institute on Alcohol Abuse and Alcoholism, National Institutes of Health, Rockville, MD, United States; 3 Department of Psychology, Indiana University - Indianapolis, Indianapolis, IN, United States

**Keywords:** anxiety, GPR110, hippocampus, learning and memory, proteomics

## Abstract

**Introduction:**

The adhesion G Protein-Coupled Receptor 110 (GPR110) is highly expressed in brain during development. Although its level is broadly reduced in adulthood, GPR110 expression notably persists in the hippocampus, yet its role in adult neurobehavioral function remains incompletely defined. In this study, we investigated whether altering GPR110 levels affects behavior and associated molecular pathways in adult mice.

**Methods:**

Using mice with global deletion of GPR110 and mice with Cre-dependent overexpression of GPR110 in glutamatergic neurons, we performed behavioral testing and transcriptomic and proteomic analyses of hippocampal and cortical tissue.

**Results:**

Global deletion of GPR110 led to increased immobility in the open field test and impaired learning and memory, accompanied by downregulation of genes and proteins involved in synaptogenesis, neurotransmission, glutamatergic signaling, and neuronal development. In contrast, overexpression of GPR110 selectively in glutamatergic neurons reduced anxiety- and compulsive-like behaviors with enhanced receptor-mediated signaling and proteins linked to neuronal morphogenesis and synaptic communication.

**Discussion:**

Together, these findings demonstrate that GPR110 regulates anxiety-related and cognitive behaviors in adult mice and modulates synaptic and signaling pathways that support neuronal structure and communication. The GPR110-dependent mechanism as a new target for neurobehavioral modulation may provide a strategy to mitigate anxiety- and cognition-related pathophysiologic behavioral conditions.

## 1 Introduction

The adhesion G Protein-Coupled Receptor 110 (GPR110) is a developmentally active receptor involved in neurogenesis, axonogenesis, and synaptogenesis. Its expression in the brain diminishes after development and in adulthood it is primarily found in the hippocampus where neurogenesis remains active throughout the life span (Lee et al., 2016). GPR110 expression, however, increases in response to insult or injury, promoting neuritogenesis and synaptogenesis while reducing neuroinflammation (Lee et al., 2016; Park et al., 2019; Banerjee et al., 2023).

GPR110 is activated by synaptamide (*N*-docosahexaenoylethanolamine), a metabolite of the omega-3 fatty acid docosahexaenoic acid (DHA). Binding of synaptamide to GPR110 activates the Gα signaling cascade, leading to the increased production of cAMP and phosphorylation of PKA/CREB, a mechanism critical for neuronal survival and repair (Huang et al., 2020; Lee et al., 2016). After traumatic brain injury (Chen et al., 2021) or optic nerve injury (Kwon et al., 2021, 2023), activation of GPR110 by synaptamide mitigates the loss of retinal ganglion cells and reduces microgliosis, astrogliosis, and axonal degeneration. The receptor’s role in reducing neuroinflammation is particularly significant, as chronic inflammation is a common feature of many neurodegenerative diseases. Synaptamide suppresses lipopolysaccharide (LPS)-induced inflammatory responses in microglia and peripheral innate immune cells through a GPR110/cAMP-dependent manner (Park et al., 2019). DHA supplementation, which elevates the endogenous level of synaptamide through metabolism, increases the expression of glutamatergic receptors without affecting GABA receptor levels, improving long-term potentiation and synaptic activity (Cao et al., 2009).

Previous work has highlighted the importance of GPR110 in learning and memory, demonstrating that global GPR110 knockout (KO) mice exhibit impaired performance in the novel object recognition and the Morris water maze tests (Lee et al., 2016). However, the study has been limited to male mice, and the role of GPR110 in other behavioral domains remains unexplored. Moreover, there has been a lack of research on the effects of GPR110 overexpression in healthy adult brains, although GPR110 expression increases following insult and injury.

Here, we address these gaps by examining both global loss of GPR110 and targeted overexpression of GPR110 in VGluT2-expressing glutamatergic neurons in both male and female mice. We assessed learning, memory, anxiety-like behavior, and compulsive-like behavior across both models and paired these behavioral findings with hippocampal and cortical transcriptomic and proteomic analyses. We found in this study that altering GPR110 expression levels modulates neurobehavioral outcomes through changes in GPR110-associated molecular networks.

## 2 Methods

### 2.1 Animals

Adult (12–16 weeks) male and female GPR110 wildtype (WT) and knockout (KO) mice were used for the experiments. GPR110 KO and WT mice were generated from GPR110 heterozygous mice on a C57Bl/6N genetic background. The heterozygous mice were obtained from the Knockout Mouse Project (KOMP) Repository (MMRRC_046507-UCD). Heterozygous males and females were bred to produce WT and KO offspring.

The pCAG-Lox-STOP-Lox-mouse *Adgrf1*-IRES-tdTomato-bGHpolyA was inserted into the C57Bl/6 J mouse *ROSA26* locus to create the GPR110 KI mouse (Jackson Laboratories). To overexpress GPR110 in glutamatergic neurons, GPR110 KI mice were crossed with VGluT2-ires-Cre mice (*Slc17a6^tm2(cre)Lowl^/J.* Jax stock #016963, Vong et al., 2011). In the GPR110 KI line, tdTomato expression is Cre-dependent and serves as a reporter of recombination. GPR110 KI mice not crossed with a Cre mouse (GPR110 KI-WT) were used as control. Mice were group housed (2–4 mice per cage) on a 12-h light/dark cycle, with lights on at 6:30 a.m. Food (Envigo, 7,017) and water were provided *ad libitum.* All experiments were carried out in accordance with the guiding principles for the care and use of animals approved by the Animal Care and Use Committee (ACUC) at the National Institute on Alcohol Abuse and Alcoholism (LMS-HK-13).

### 2.2 Behavioral setup

To assess the role of GPR110 in learning, memory, and anxiety-like behaviors, a battery of tests was conducted. Prior to each behavioral experiment, mice were acclimated to the experimental room for at least 30 min in their home cages. Testing began at 10 a.m. in a well-lit (~400 lux) behavior room. A minimum rest period of 2 days was given between tests. All behavioral tests were analyzed using AnyMaze software (7.4). Throughout testing, the experimenter sat quietly in the corner of the room.

### 2.3 Open field test

Anxiety-like behaviors were assessed using the open field test. The apparatus (40 cm × 40 cm) had a grey base and black walls (Stoelting). The center zone was defined as a 20 cm × 20 cm square in the middle of the arena. Mice were placed in the apparatus for 5 min, and the time spent in the center, time immobile, and total distance traveled were measured. Increased time in the center was interpreted as reduced anxiety, while increased immobility indicated heightened anxiety.

### 2.4 Novel object recognition test (NORT)

Learning and memory were assessed using NORT. NORT was conducted using the same 40 cm × 40 cm open field apparatus. The test consisted of three phases. During the habituation phase, mice freely explored the empty apparatus for 5 min. Data from this phase was used to assess baseline anxiety-like behaviors as described above. In the familiarization phase, two identical objects were placed in the arena, and mice were allowed to explore them freely for 10 min. The objects (pyramid, dome, steps, icosahedron) were 3D-printed in-house based on recommendations from Inayat et al., 2021. Lastly, 2 h later, during the testing phase, one familiar object was replaced with a novel object, and mice were allowed to explore for 10 min. Object exploration was defined as the mouse’s nose being within 1 cm of the object, including instances where the center of the mouse was positioned on top of the object. Mice were excluded from the final analysis if their total exploration time across both objects during the familiarization phase was less than 20 s.

### 2.5 Marble burying test

The marble burying test was conducted to assess compulsivity-like behavior. Testing took place in cages resembling the home cages, filled with enough bedding to fully cover the marbles. Fourteen marbles were arranged in two columns of seven, spaced 4 cm apart. Mice were placed at the edge of the cage and allowed to freely explore for 30 min. After the test, the mice were carefully returned to their home cage. Photos of the marble arrangement were taken from the top and two sides. Two experimenters, blinded to experimental conditions, independently counted the number of marbles buried at least halfway. A higher number of buried marbles indicated increased compulsivity-like behaviors.

### 2.6 Brain dissection

Following the behavioral experiments, the mice were anesthetized with isoflurane and transcardially perfused with 1× cold PBS to clear blood from the brain. The mice were then decapitated, and the brains were dissected out. For mass spectrometry the hippocampus or cortex was isolated bilaterally. The hippocampi and cortices were quickly placed in a microcentrifuge tube and snap frozen in liquid nitrogen before transferring to storage at −80 °C. For RNA sequencing, the cortex, including the hippocampus, was isolated from half hemisphere and snap frozen. For RT-PCR, half hemisphere was cut out and snap frozen. For examining TdTomato expression, the brains were sliced at 40um thickness on Leica VT 1200 vibratome, mounted on slides, and coverslipped with a DAPI mounting medium. Imaging was conducted on a Zeiss LSM 880 confocal microscope.

### 2.7 Real-time RT-PCR

Tissue was lysed and homogenized in TRIzol Reagent (ThermoFisher Scientific, Cat. No. 15529026) and reverse transcribed to cDNA using First Strand cDNA Synthesis Kit (Syd Labs). Expression of GPR110 and GAPDH was measured using SYBR Green qPCR Master Mix/Low ROX (Syd Labs) assay. The resulting first strand cDNA was used for the PCR analysis in Agilent MX3005P instrument. Triplicates were done for each assay. The relative expression of mRNA was calculated after normalization to GAPDH mRNA. Primer sequences are indicated in Supplementary Table 1.

### 2.8 Protein extraction for proteomics

Hippocampal or cortical tissues were placed in 2 mL microtubes containing 1.4 mm ceramic beads (Omni International Cat. No. 19–627). Cell lysis buffer (Cell Signaling Tech. Cat. No. 9803) supplemented with Halt protease and phosphatase inhibitors (ThermoFisher Scientific, Cat. No. 78428 and 78429) was added at a ratio of 10 μL per mg of tissue. Samples were homogenized for 30 s at a speed of 4.85 using a Ruptor 12 homogenizer (Omni International). The homogenates were centrifuged at 10,000 g for 10 min at 4 °C, after which the supernatant containing soluble proteins was collected. Protein concentration was determined using the BCA Protein Assay Kit (Thermo Fisher Scientific, Cat. No. 23225) according to the manufacturer’s instructions.

### 2.9 SDS-PAGE and in-gel digestion

The immunopurified proteins were incubated in 4× NuPage lithium dodecyl sulfate (LDS) sample buffer (Invitrogen, Cat. No. NP0007) with 5% mercaptoethanol for 30 min at 37 °C. The samples (40 μg) were then loaded into 10-well NuPAGE 4–12% Bis-Tris Mini Protein Gels (Invitrogen, Cat. No. NP0323BOX). Electrophoresis was carried out at a constant voltage of 200 V for 30–60 min using MOPS SDS running buffer (Invitrogen, Cat. No. NP0001). The proteins were stained with SimplyBlue SafeStain (Invitrogen, Cat. No. LC6060) for 1 h at room temperature. After rinsing with ddH2O the gel was incubated with ddH2O overnight at 4 °C.

For in-gel digestion, the entire gel was cut to 10 fractions. Each fraction was diced into 1–2 mm pieces. To remove the stain, the gel pieces were washed twice with 25 mM NH_4_HCO_3_ in 50% acetonitrile (ACN), shaking for 30 min each, followed by 100% ACN for 1–2 min. The gel pieces were dried by vacuum centrifugation and subjected to in-gel reduction with 10 mM dithiothreitol (DTT) at 56 °C for 1 h and alkylation with 110 mM iodoacetamide at room temperature for 45 min in the dark. The gels were then sequentially washed once with 25 mM NH_4_HCO_3_, twice with 25 mM NH_4_HCO_3_/50% ACN, once with 100% ACN. The gel pieces were then dried by vacuum centrifugation, rehydrated on ice with 12.5 ng/mL trypsin (Promega Cat. no. V5072) in 25 mM NH_4_HCO_3_ for 30 min. The digestion continued overnight at 37 °C. The peptides were then extracted in 5% formic acid/50% ACN, concentrated by vacuum centrifugation, and desalted using C-18 ziptip (Millipore, Cat. No. ZTC18S096) according to the manufacturer’s instructions.

### 2.10 Nano-HPLC MS/MS analysis

Nano-LC-ESI-MS/MS was performed on an Orbitrap Fusion™ Lumos™ Tribrid™ mass spectrometer (Thermo Scientific) equipped with an UltiMate 3,000 RSLCnano system. The mobile phase consisted of 0.1% formic acid (solvent A) and 0.1% formic acid in 98% ACN (solvent B). Peptide samples were loaded into a C18 trap column (Acclaim PepMap100 C18, Thermo Scientific) and separated by a 15-cm Acclaim RSLC column (Thermo Scientific) at a flow rate of 300 nL/min with a gradient from 5 to 35% solvent B in 95 min. LC eluent was sprayed into the mass spectrometer via a stainless-steel emitter (Thermo Scientific) with a spray voltage of 2.2 kV in positive-ion mode. Full scan spectra from *m/z* 350–1700 at resolution of 120,000 were acquired in the Orbitrap. Data-dependent MS/MS spectra were acquired in the ion trap using collision-induced dissociation (CID) with a normalized energy of 30. Other parameters included an exclusion duration of 30 s, mass tolerance of 10 parts per million (ppm), and fragmentation of charge states 2–6.

### 2.11 Protein identification

The MS/MS data was searched against the NCBIprot_mouse database using Mascot Distiller (v2.8.3). Search parameters included trypsin (Enzyme), 10 ppm (precursor ion mass tolerance), 0.3 Da (fragment ion mass tolerance), 2 (maximum missed cleavages allowed), carbamidomethyl of cysteine residues (fixed modification), and oxidation of methionine (variable modification). The criteria used to filter data included 1% false positive rate and Expect value of less than 0.05 for significant peptide matches.

### 2.12 Protein quantitation and pathway analysis

Mass spectrometric data from biological replicates were analyzed using Progenesis QI for Proteomics software (Waters) for label-free protein quantitation, as described previously (Huang and Kim, 2023). Briefly, raw MS/MS data were imported, chromatograms were automatically aligned, peak lists were generated with the default algorithm, and normalization was performed based on the assumption that most features remain unchanged across all samples. Protein quantitation was performed using non-conflicting features. Proteins showing at least a 1.15-fold change with a *p*-value of less than 0.05 were imported into Ingenuity Pathway Analysis (Qiagen, IPA) and subjected to Core Analysis.

### 2.13 Transcriptomics

Two RNA sequencing analyses were conducted, first with 6 WT and 6 GPR110 KO cortex and hippocampus samples and second with 6 WT and 6 GPR110 KI-VGluT2 cortex and hippocampus samples. The data were collected and mapped to reference genome, the gene expression was quantified, followed by differential expression and functional analyses. The resulting *p*-values were adjusted using the Benjamini and Hochberg’s approach for controlling the False Discovery Rate (FDR). Genes with the adjusted *p*-value of <0.05 were considered differentially expressed. To gain insight into biological functions and pathways associated with the differentially expressed genes, Gene Ontology (GO) enrichment analyses were conducted.

### 2.14 Data collection and statistical analysis

Behavioral data was analyzed using Microsoft Excel 365 and GraphPad Prism 10.3. Outliers were identified using ROUT method at *Q* = 5% and were excluded from analysis. Groups were compared using unpaired *t*-test. *p*-values below 0.05 were considered significant.

## 3 Results

### 3.1 GPR110 knockout mice exhibit an increase in specific anxiety-like behavior and worsened learning and memory

**Figure 1:**
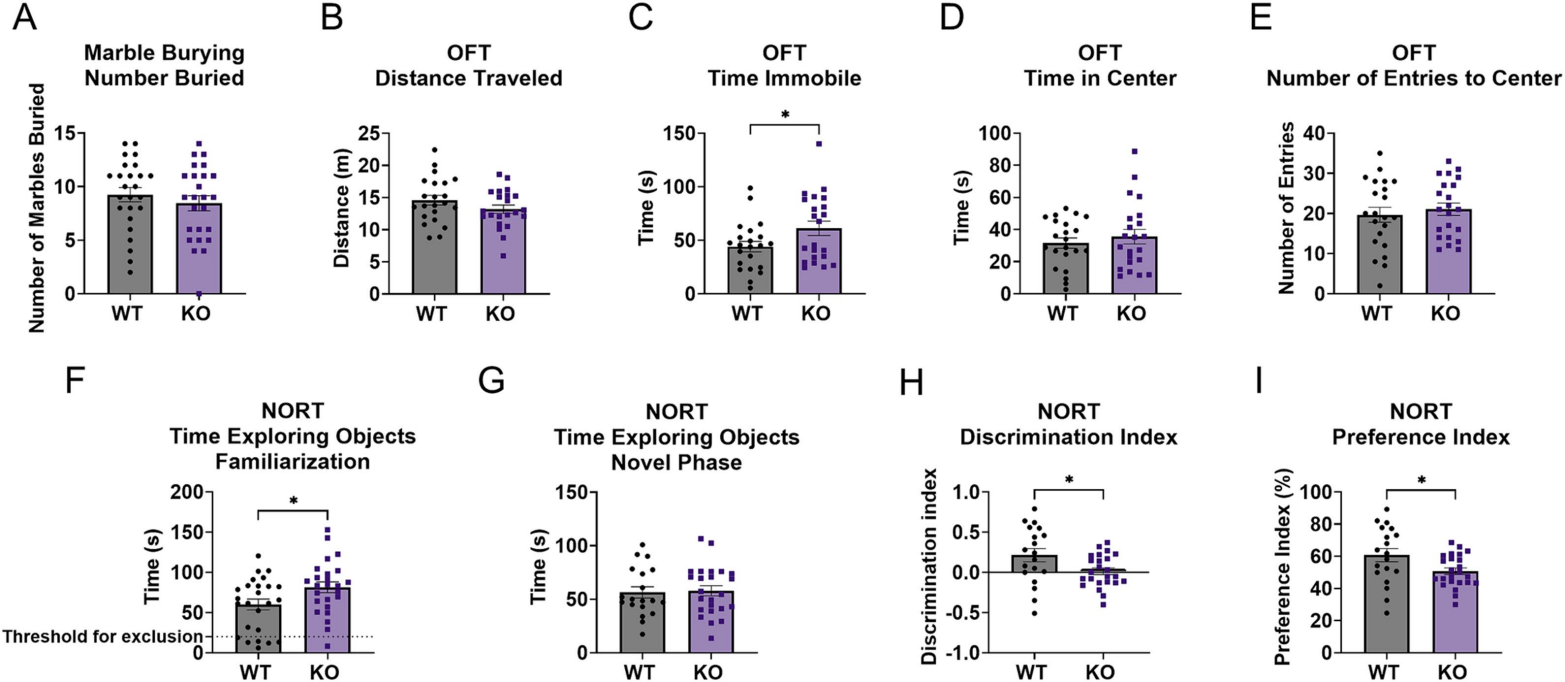
Effects of GPR110 KO on behavioral outcome. GPR110 WT and KO mice underwent the marble burying test **(A)**, the open field test (OFT, **B–E**), and the novel object recognition test (NORT, **F–I**). A total of 50 mice were used for the behavioral experiments, 6 of which were excluded from OFT due to being identified as outliers and 7 excluded from NORT due to spending less than 20 s exploring objects in the familiarization phase. * Describes significant difference at *p* < 0.05.

A total of 50 mice were used for the marble burying and the open field tests. There were no significant differences in the number of marbles buried between the WT and GPR110 KO groups (Figure 1A, *p* = 0.4095, *t* = 0.8320, df = 48). GPR110 KO mice spent increased time immobile compared to WT mice (Figure 1C, *p* = 0.0449, *t* = 2.067, df = 42), with no significant differences in distance traveled (Figure 1B, *p* = 0.1753, *t* = 1.379, df = 42), time spent in center (Figure 1D, *p* = 0.4735, *t* = 0.7233, df = 42), or number of entries to center (Figure 1E, *p* = 0.5618, *t* = 0.5848, df = 42).

GPR110 KO mice had increased time spent exploring both objects in the familiarization phase of the novel object test (Figure 1F, *p* = 0.0279, *t* = 2.268, df = 48). A total of 7 mice were excluded from further analysis as they spent less than 20 s with the identical objects. Time spent exploring the objects in the novel phase was not different between the groups (Figure 1G, *p* = 0.8284, *t* = 0.2181, df = 41). GPR110 KO mice had worsened discrimination index (Figure 1H, *p* = 0.0264, *t* = 2.304, df = 41) and preference index (Figure 1I, *p* = 0.0258, *t* = 2.313, df = 41) compared to WT controls.

Overall, GPR110 KO mice have increased certain anxiety-like behaviors such as immobility and have worsened learning and memory as shown by decreased discrimination index in the NORT despite increased time examining objects.

### 3.2 GPR110 KO downregulates important proteins for neurodevelopment

To elucidate the molecular and cellular mechanisms associated with the behavioral phenotypes observed in GPR110 KO mice, particularly learning and memory deficit, we analyzed hippocampal protein expression differences between WT and KO mice using mass spectrometry–based proteomics coupled with pathway analysis. We identified over 6,300 proteins from hippocampus, with 330 of these proteins showing significant changes in WT vs. KO (*p* < 0.05, fold change ≥ 1.15, *n* = 4 WT, *n* = 3 KO). Of these 330 proteins, 232 were downregulated and 98 were upregulated in GPR110 KO hippocampus (Supplementary Figure S1).

**Figure 2:**
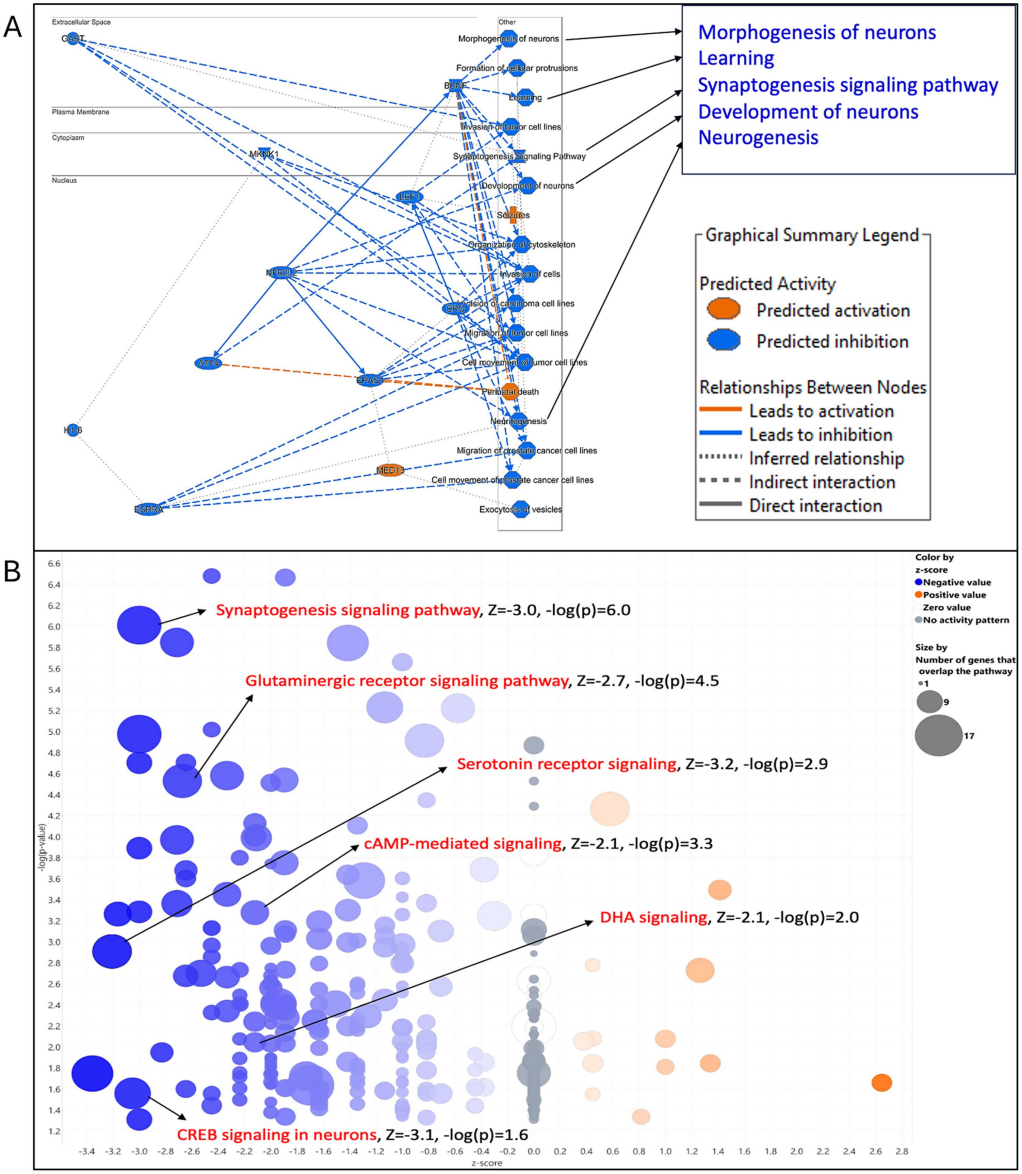
Physiological and cellular functions predictably inhibited by the changes of protein expression in GPR110 KO mice. Functional **(A)** and canonical pathway **(B)** analyses were performed with Ingenuity Pathway Analysis (IPA). *Z*-scores of ≥2 or ≤ −2 are considered significant.

To determine whether these changes reflected disruptions in specific neuronal functions, we performed pathway analysis which revealed that numerous proteins downregulated in GPR110 KO mice participate in synaptogenesis and glutamatergic receptor signaling (Table 1, Figure 2A). These proteins include several kinases (e.g., Akt, mitogen-activated protein kinase 1, phosphoinositide-3-kinase), key enzymes (e.g., adenylate cyclase 2, glutamate–ammonium ligase), transporters, ion channel receptors (such as glutamate ionotropic receptors), and other synaptic components. In addition, serotonin receptor signaling, cAMP-mediated signaling, DHA signaling, and CREB signaling were inhibited by the downregulated proteins (Figure 2B). Functional analysis indicates that these decreases are associated with impaired neuronal morphogenesis and development, synaptogenesis, neurogenesis, and learning (Figure 2A). Notably, reduced expression of these proteins in GPR110 KO mice correlates with deficits in behavioral functions, including cognition, learning, and anxiety-like behaviors, supporting the observed impairments in learning and memory (Table 1, Supplementary Figure S2).

**Table 1:** Hippocampal proteins involved in synaptogenesis signaling^1^ and glutamatergic receptor signaling^2^ pathways that were downregulated in GPR110 KO mice.

Symbol	Entrez gene name	Expr fold change	Location	Type(s)
ADCY2^1,2^	Adenylate cyclase 2	−1.407	Plasma membrane	Enzyme
AKT1^1,2^	AKT serine/threonine kinase 1	−1.170	Cytoplasm	Kinase
AP2S1^1^	Adaptor related protein complex 2 subunit sigma 1	−1.174	Cytoplasm	Transporter
ARPC4^1^	Actin related protein 2/3 complex subunit 4	−1.184	Cytoplasm	Other
EPHB1^1^	EPH receptor B1	−1.396	Plasma membrane	Kinase
FARP1^1^	FERM, ARH/RhoGEF and pleckstrin domain protein 1	−1.250	Plasma membrane	Other
GLS^2^	Glutaminase	−1.373	Cytoplasm	Enzyme
GLUL^2^	Glutamate-ammonia ligase	−1.256	Cytoplasm	Enzyme
GRIK2^2^	Glutamate ionotropic receptor kainate type subunit 2	−1.268	Plasma membrane	Ion channel
GRIK5^2^	Glutamate ionotropic receptor kainate type subunit 5	−1.443	Plasma membrane	Ion channel
LYN^1^	LYN proto-oncogene, Src family tyrosine kinase	−2.131	Cytoplasm	Kinase
MAPK1^1,2^	Mitogen-activated protein kinase 1	−1.236	Cytoplasm	Kinase
MAPT^1^	Microtubule associated protein tau	−1.253	Plasma membrane	Other
PIK3R2^1,2^	Phosphoinositide-3-kinase regulatory subunit 2	−1.522	Cytoplasm	Kinase
PLCB1^2^	Phospholipase C beta 1	−8.547	Cytoplasm	Enzyme
PLCH2^2^	Phospholipase C eta 2	−1.277	Cytoplasm	Enzyme
PPP3CB^2^	Protein phosphatase 3 catalytic subunit beta	−1.271	Plasma membrane	Phosphatase
RAB3A^1^	RAB3A, member RAS oncogene family	−1.320	Cytoplasm	Enzyme
SLC1A2^2^	Solute carrier family 1 member 2	−1.249	Plasma membrane	Transporter
SYN2^1^	Synapsin II	−1.257	Plasma membrane	Other
SYT1^1^	Synaptotagmin 1	−1.593	Cytoplasm	Transporter
WASF1^1^	WASP family member 1	−1.272	Nucleus	Other

^1^Synaptogenesis signaling. ^2^Glutamatergic receptor signaling.

### 3.3 GPR110 KO leads to the downregulation of genes related to neurodevelopment and neurotransmission

**Figure 3:**
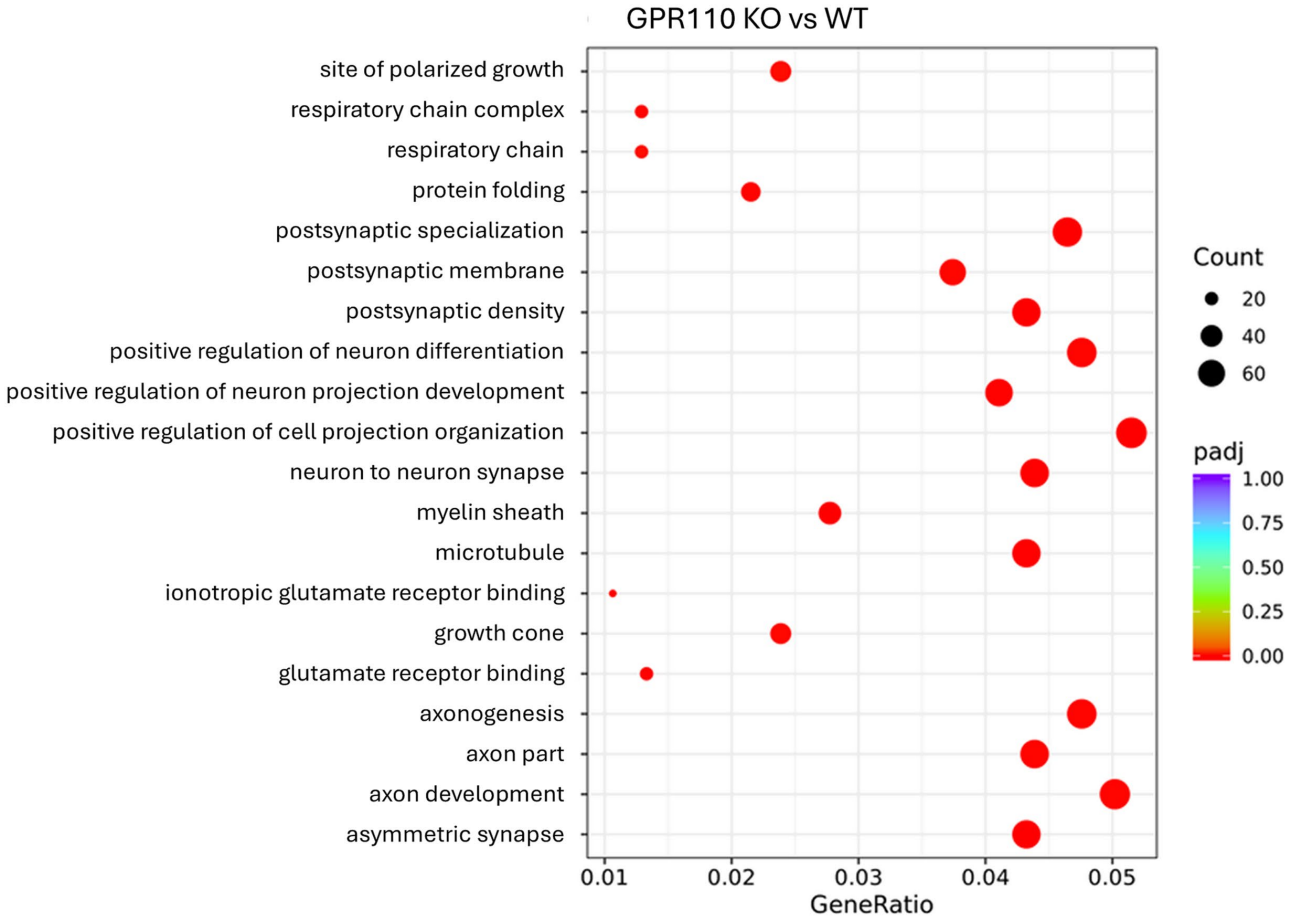
RNA sequencing analysis of differentially expressed genes. GO enrichment analysis of top 20 significantly enriched pathways. The red color represents adjusted *p*-value of 0.00, while the size of the dot represents the number of DEGs. The gene ratio is KO over WT.

A total of 29,735 differentially expressed genes were identified by RNA sequencing, of which 1861 were significantly different between the groups (*p* < 0.05, |log2FoldChange| > 0, *n* = 6 WT, *n* = 6 KO). Of these 1861, 922 were upregulated and 939 were downregulated. The GO analysis revealed downregulation in biological processes such as postsynaptic specialization, postsynaptic membrane, postsynaptic density, neuron to neuron synapse, glutamate receptor binding, axonogenesis, and axon development (Figure 3). These findings complement the proteomic findings of downregulation of pathways involved in neurodevelopment and neurotransmission.

### 3.4 GPR110 KI leads to overexpression of GPR110 in the VGluT2 cells in the brain

**Figure 4:**
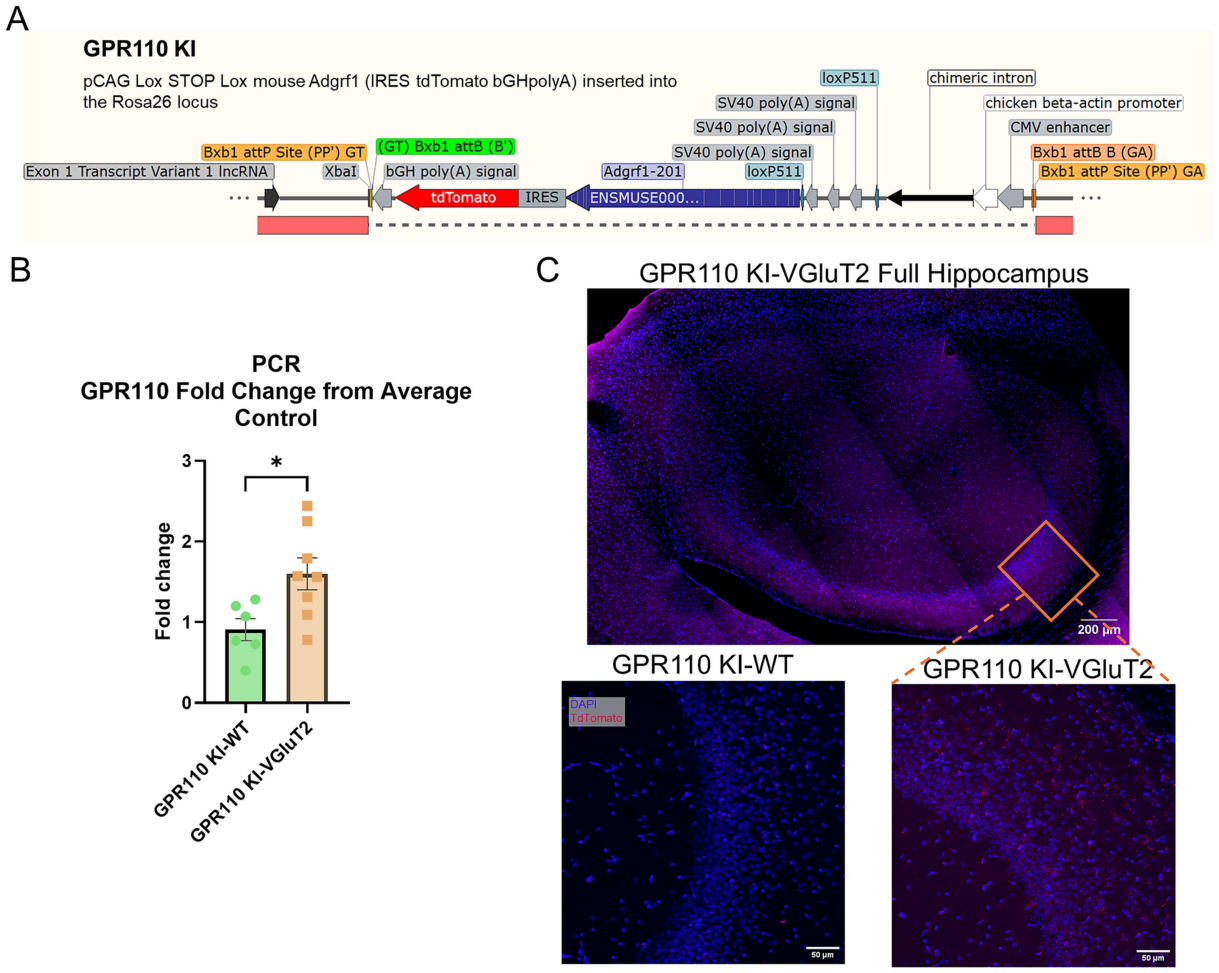
**(A)** Gene modification for GPR110 knockin (KI) representative sequence. **(B)** Fold change of GPR110 expression compared to the average expression of WT controls. Outlier test resulted in the exclusion of one WT data point. **(C)** GPR110 expression in GPR110 KI-VGluT2 hippocampus visualized by TdTomato (red) and with DAPI contrast (blue). * describes significant difference at *p* < 0.05.

Since GPR110 KO mice exhibited behavioral deficits, we examined the effect of GPR110 overexpression on behavioral outcomes. For this purpose, we crossed GPR110 KI mice with VGluT2-IRES-Cre mice (GPR110 KI x VGluT2, Figure 4A). The GPR110 KI-VGluT2 overexpression was validated in the offspring of GPR110 KI x VGluT2 mice by RT-qPCR (*p* = 0.0206, *t* = 2.665, df = 12, Figure 4B, *n* = 6 GPR110 KI-WT, *n* = 8 GPR110 KI-VGluT2) and TdTomato expression, which served as a reporter of Cre-mediated recombination (Figure 4C).

### 3.5 GPR110 overexpression in VGluT2 cells reduces compulsive- and anxiety-like behaviors

**Figure 5:**
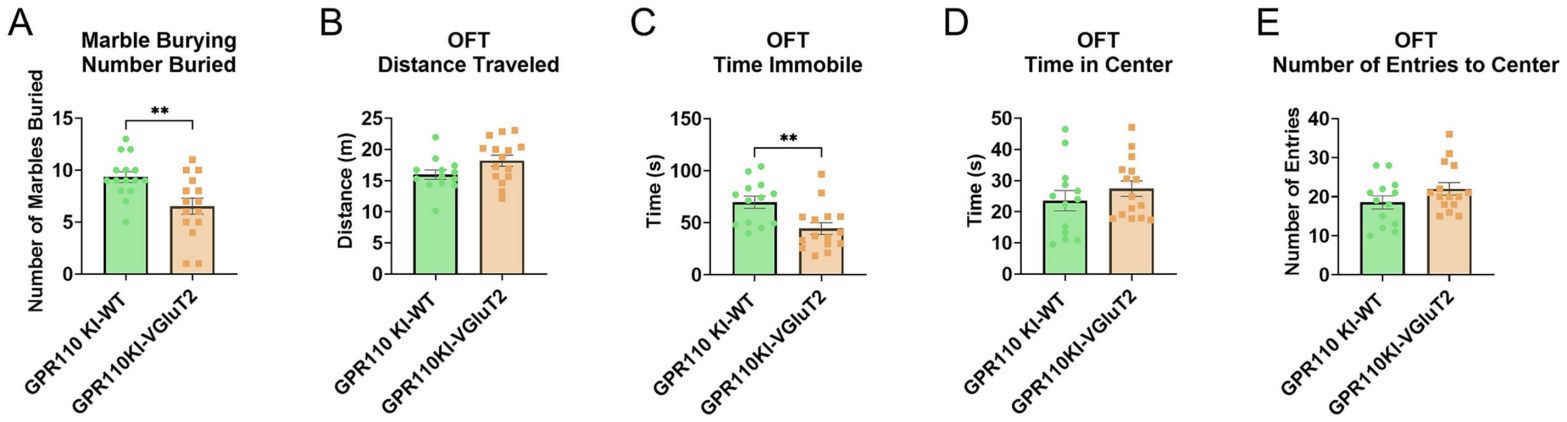
Effects of GPR110 overexpression in VGluT2 neurons on behavioral outcome. GPR110 KI-WT and GPR110 KI-VGluT2 mice underwent the marble burying test **(A)** and the open field test (OFT, **B–E**). * describes significant difference at *p* < 0.05.

To assess the role of GPR110 overexpression in behavioral outcomes, we used the GPR110 KI-VGluT2 mice for compulsive-like and anxiety-like behaviors. A total of 30 mice were used for both the marble burying and the open field tests. In the marble burying test, we found significant reduction in the marbles buried by the GPR110 KI-VGluT2 mice compared to wildtype (GPR110 KI-WT) mice (Figure 5A, *p* = 0.0059, *t* = 2.983, df = 28). In the open field test, we found a significant decrease in time spent immobile in the GPR110 KI-VGluT2 mice compared to GPR110 KI-WT (Figure 5C, *p* = 0.0047, *t* = 3.092, df = 26). No significant differences were found in the other measures (distance traveled: Figure 5B, *p* = 0.0689, *t* = 1.898, df = 26; time in center: Figure 5D, *p* = 0.3467, *t* = 0.9584, df = 26; number of entries to center: Figure 5E, *p* = 0.1490, *t* = 1.487, df = 26). While there were no significant differences in the time spent in center of the OFT, the decreased immobility and decreased number of marbles buried suggest that GPR110 overexpression in glutamatergic neurons does reduce compulsive-like and anxiety-like behaviors.

### 3.6 GPR110 overexpression in VGluT2 neurons enhances receptor-mediated signaling linked to synaptic communication

**Figure 6:**
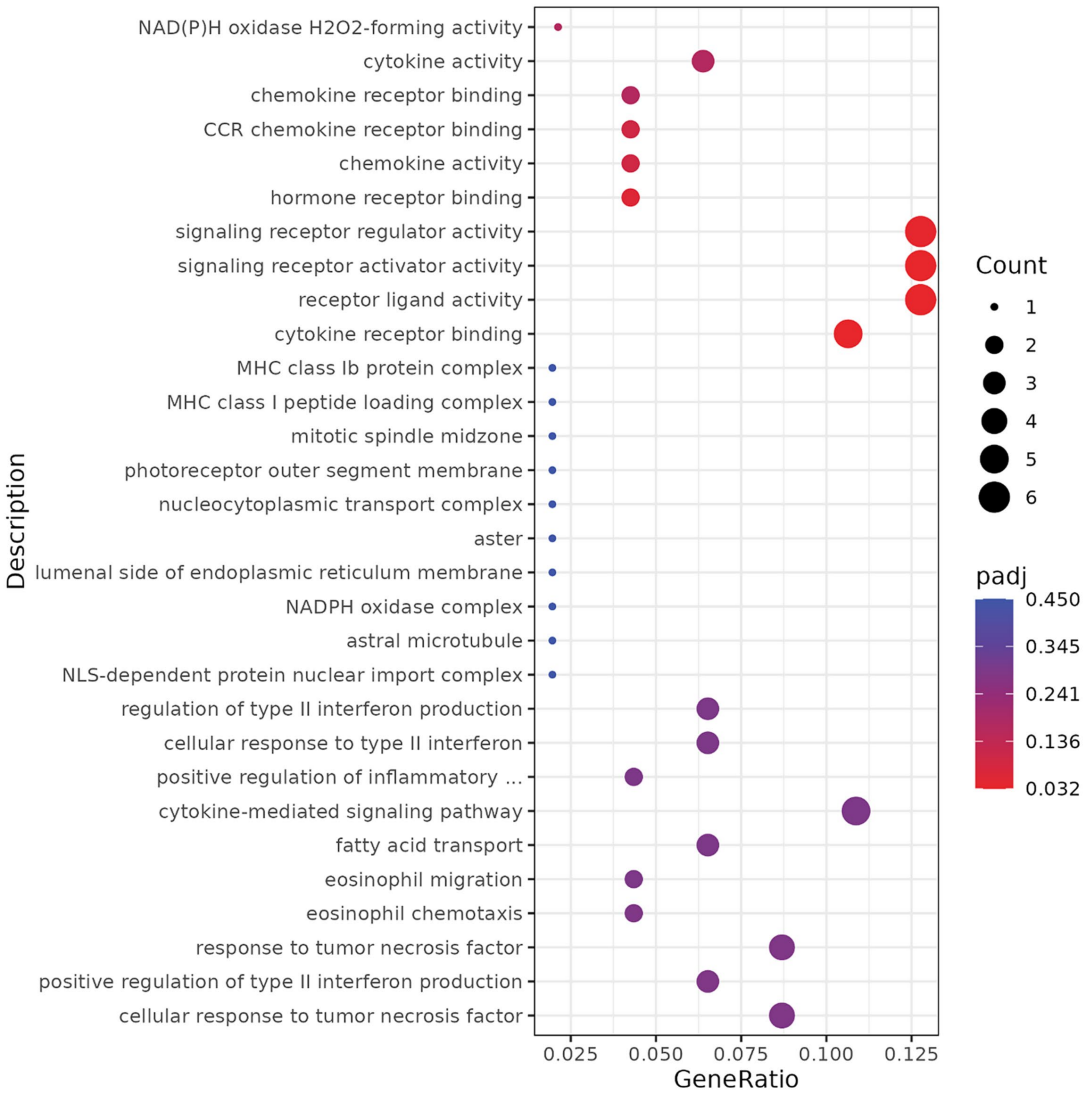
RNA sequencing analysis of differentially expressed genes upregulated in GPR110 KI-VGluT2 compared to GPR110 KI-WT. GO enrichment analysis of top 30 enriched pathways. The red color represents adjusted *p*-value of 0.032, while the size of the dot represents the number of DEGs.

To complement the GPR110 KO RNA-seq findings, we examined transcriptional changes in the cortex and hippocampus resulting from GPR110 overexpression in VGluT2-expressing neurons. Differential expression analysis identified 115 upregulated and 304 downregulated transcripts (*p* ≤ 0.05 and |log₂FoldChange| ≥ 1) (Figure 6, *n* = 6 GPR110 KI-WT, *n* = 6 GPR110 KI-VGluT2). GO enrichment of upregulated transcripts in GPR110 KI–VGluT2 mice highlighted pathways related to receptor-mediated signaling, including ligand–receptor interactions, cytokine receptor binding, and chemokine activity (Figure 6).

Mass spectrometric analysis of the cortex of GPR110 KI-VGluT2 mice compared to GPR110 KI-WT identified over 6,500 proteins, with 89 proteins being significantly changed between GPR110 KI-WT and GPR110 KI-VGluT2 (*p* < 0.05, fold change ≥ 1.15, *n* = 4 GPR110 KI-WT, *n* = 5 GPR110 KI-VGluT2). Of the 89, 41 were downregulated and 48 were upregulated in GPR110 KI-VGluT2 cortex. In contrast to the case with GPR110 KO, some proteins involved in glutamatergic activity, synaptic signaling, and neuronal plasticity were upregulated in the cortex of GPR110 KI-VGluT2 mice compared to GPR110 KI-WT (Supplementary Table S1). The IPA analysis showed an upregulation of proteins involved in neuritogenesis, morphogenesis, and cellular assembly in pathways associated with cellular assembly and organization (Supplementary Figure S3) including multiple cytoskeletal regulators (e.g., doublecortin, CAMSAP2), structural intermediate filaments (e.g., NEFL, INA), and signaling scaffolds (e.g., AKAP12) (Table 2). Together with the synaptic and glutamatergic deficits observed in the GPR110 KO, these findings suggest that GPR110 contributes to pathways that enable synaptic organization and plasticity.

**Table 2:** Cortical proteins involved in cellular assembly and organization that were upregulated in GPR110 KI-VGluT2 mice.

Symbol	Entrez gene name	Expr fold change
DCX	Doublecortin	1.805
CAMSAP2	Calmodulin regulated spectrin associated protein family member 2	1.159
TPM2	Tropomyosin-2	6.871
DAAM1	Disheveled-associated activator of morphogenesis 1	1.172
AKAP12	A-kinase anchor protein 12	1.545
BCL2	B-cell lymphoma 2 apoptosis regulator	1.457
NEFL	Neurofilament light chain	1.236
INA	Internexin neuronal intermediate filament protein alpha	1.166

## 4 Discussion

In this study, we show that loss of GPR110 leads to increased immobility in the open field test and impaired learning and memory in mice, while GPR110 overexpression reduces compulsive- and anxiety-like behaviors. At the molecular level, we found that genes and proteins involved in neurotransmission, synaptogenesis, and receptor signaling were altered by the manipulation of GPR110 expression. These findings demonstrate that GPR110 contributes to adult anxiety-like behavior and cognitive function likely by altering synaptic and signaling proteins relevant to these behavioral outcomes.

A major contribution of this work is the characterization of GPR110 knockout (KO) mice, which revealed clear behavioral and molecular consequences of losing GPR110 function. GPR110 KO mice showed increased immobility in the open field test and impaired learning and memory in the NORT (Figure 1), consistent with heightened anxiety-like behavior and reduced cognitive performance. Notably, the increase in anxiety-like behavior was specific to immobility, with no changes observed in center time or overall locomotion, suggesting that the behavioral consequences of GPR110 loss may be restricted to particular components of anxiety-related behavior. Likewise, marble burying behavior was not altered in KO mice, indicating that GPR110 deletion does not broadly affect all anxiety- or compulsive-like behaviors but may influence only select behavioral domains. These behavioral changes align with our transcriptomic and proteomic findings: the KO hippocampus exhibited broad downregulation of genes and proteins involved in neurogenesis, synaptogenesis, glutamatergic receptor signaling, and neurotransmission (Figures 2, 3). These results fill an important gap in the literature by demonstrating that loss of GPR110 disrupts not only developmental processes, as previously reported (Lee et al., 2016), but also ongoing adult neuronal signaling pathways that support cognition and anxiety-like behavior.

Building on the findings for KO animals, we next examined how increasing GPR110 expression specifically in glutamatergic neurons affects behavior and molecular signaling. This study is the first to generate a cell type–specific GPR110 overexpression model, achieved by crossing the GPR110 knock-in strain with VGluT2-Cre mice. Although GPR110 overexpression was expected to occur from the beginning of development and likely subjected to decline with age, receptor overexpression was clearly indicated in adult stage (Figure 4B). Overexpression of GPR110 in VGluT2-expressing neurons produced a clear anxiolytic-like phenotype, as reflected by decreased immobility in the open field test and reduced marble burying (Figure 5). These behavioral changes underscore the importance of GPR110 in excitatory neurotransmission and emotional regulation. Because the overexpression was driven globally in all VGluT2-positive neurons, regional specificity could not be resolved. This is a relevant limitation, as previous studies have shown that VGluT2 neurons are involved in anxiety regulation, with region-dependent effects on behavior (Nordenankar et al., 2015; Roccaro-Waldmeyer et al., 2018; Luo et al., 2023; Goodman et al., 2024; McGovern et al., 2024). Thus, future studies employing region- or circuit-specific GPR110 manipulation may reveal stronger and more localized effects on anxiety-like behaviors.

At the molecular level, however, the effects of GPR110 overexpression were more subtle than those seen in the KO mice. Transcriptomic analysis revealed significant increases in several genes related to receptor-mediated signaling (Figure 6). Similarly, the proteome analysis of GPR110 KI-VGluT2 cortex identified proteins trending toward increased cellular structure and organization, but these pathway-level changes did not reach the same level of statistical significance observed in the KO dataset (Table 2). This pattern is not surprising as the GPR110-related molecular and structural features are most likely shaped during development where endogenous GPR110 expression in the brain is already high without overexpression. The KI-induced increase may not be large enough to cause robust proteomic shifts in otherwise healthy neurons in both developmental and adult stages. Apparently, such subtle changes may be sufficient to improve anxiety-like behaviors as clearly indicated in Figure 5.

An additional methodological consideration is that the KO and KI proteome analyses were performed in different brain regions. The KO proteomics focused on the hippocampus, which is most relevant to the learning and memory impairments observed in KO mice. In contrast, the GPR110 KI-VGluT2 proteomics were conducted in cortex to align with the anxiety-related behaviors measured in those animals. Because GPR110 shows region-specific functional roles, these tissue differences likely contributed to the distinct molecular profiles observed between models. Importantly, they also highlight the need for future studies that evaluate the effects of region- or circuit-specific manipulations of GPR110 to better understand its diverse functions.

Taken together, the KO and KI datasets provide complementary perspectives: loss of GPR110 leads to behavioral impairments and downregulation of neurodevelopmental and glutamatergic pathways, whereas increased GPR110 expression produces behavioral improvements accompanied by more modest but directionally consistent molecular changes. These findings underscore the idea that GPR110 supports neuronal function in adulthood through both structural and signaling mechanisms, and that its impact may depend on baseline receptor levels and the specific neural circuits in which it is expressed.

In conclusion, this work highlights a key role of GPR110 in shaping adult neurobehavioral outcomes. GPR110 signaling intersects with pathways involved in synaptic plasticity, glutamatergic receptor signaling, and neuronal structural organization, suggesting a broader role for this receptor in maintaining adult synaptic function. While our models do not directly represent disease states, the pathways affected by GPR110 overlap with mechanisms implicated in several disorders in which excitatory circuit function is altered. Future studies using region-specific or inducible manipulations of GPR110 will be essential for further defining its roles in physiological and pathophysiological conditions.

## Data Availability

The mass spectrometry proteomics data have been deposited to the ProteomeXchange Consortium via the PRIDE (Perez-Riverol et al., 2025) partner repository with the dataset identifier PXD074658. Any additional data will be made available upon request.
