# Supplementary material for: Effects of GPR110 expression on neurobehavioral outcomes in mice
Source: Front Neurosci. 2026 Mar 4;20:1774433. doi: 10.3389/fnins.2026.1774433 (PMC12997541; doi:10.3389/fnins.2026.1774433)
Supplement: Supplementary file 1 [file Data_Sheet_1.docx]

**Supplementary Information**

| Supplementary Table 1: Primer sequences for PCR | | |
| --- | --- | --- |
| Gene symbol | Forward Primer | Reverse Primer |
| Gapdh | TCTGGAAAGCTGTGGCGTGAT | ATGCCAGTGAGCTTCCCGTTCAG |
| Gpr110 (Adgrf1) | AGCAGCGTCTGATGGGAATG | TCTGGACTGAACACAGACACC |

Figure S1. Volcano plot of mass spectrometry-based proteomics data. Among 6300 proteins quantified, 330 proteins altered, with 232 downregulated and 98 upregulated, based on fold change 1.15 and p<0.05 (n=4 WT and n=3 KO).

**
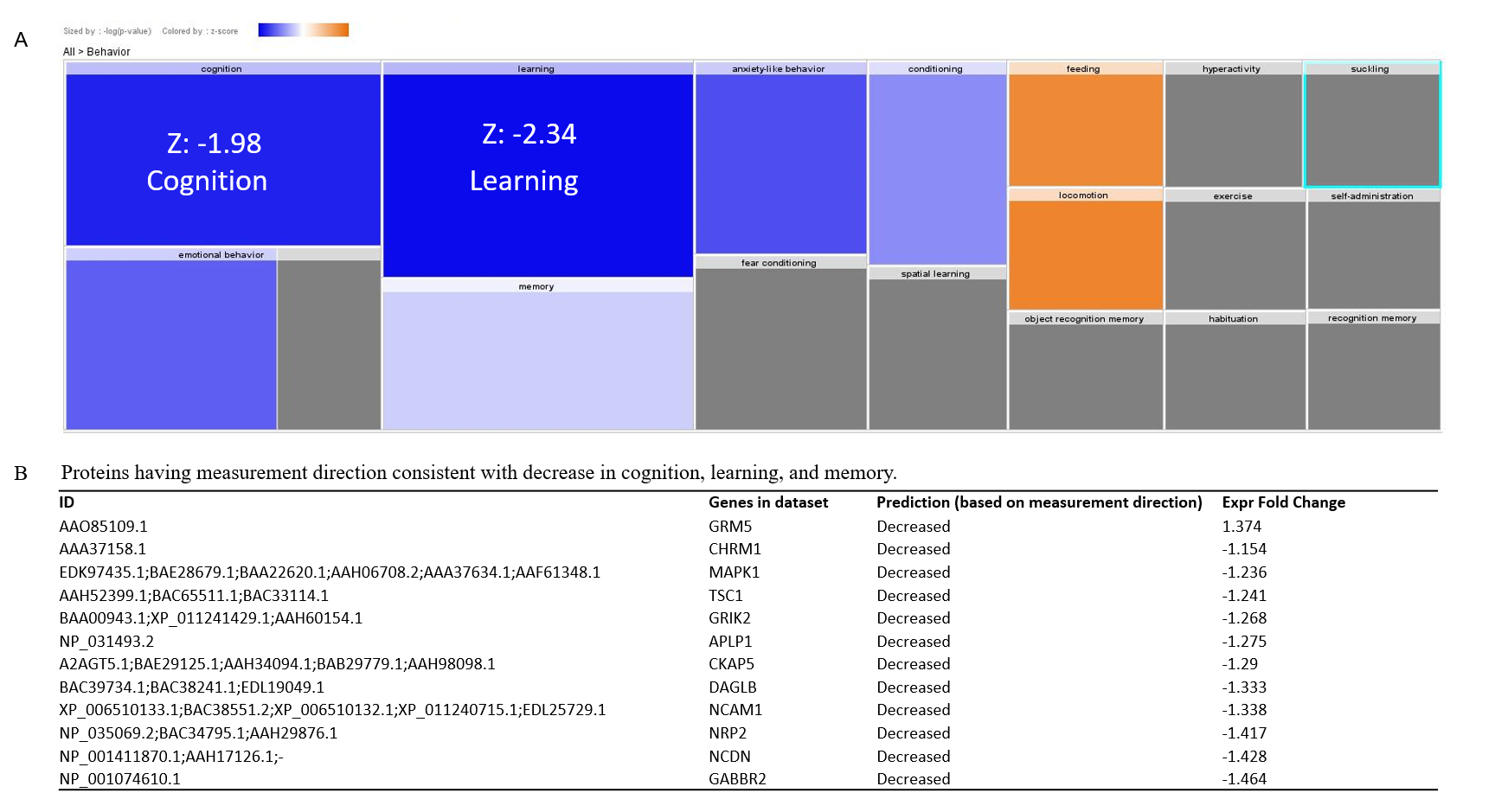
**

Figure S2. Negative effect of proteins decreased in GPR110 KO mice on behavior revealed by pathway analysis. A. Behavioral analysis by Ingenuity Pathway Analysis (IPA). B. Proteins downregulated in GPR110 KO mice are involving in cognition, learning and memory. Z-scores of ≥ 2 or ≤ -2 are considered significant.


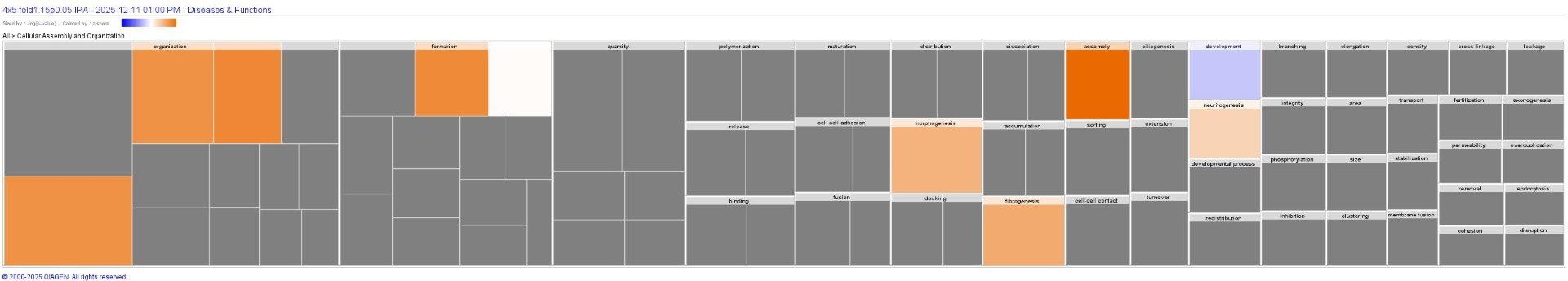


Figure S3. Analysis by Ingenuity Pathway Analysis (IPA) shows upregulation of proteins involved in Cellular Assembly and Organization in the cortex of GPR110 KI-VGluT2 mice compared to WT.

| Table S1: Cortical proteins involved in Synaptogenesis Signaling^1^ and Glutamatergic Receptor Signaling^2^ Pathways that were upregulated in GPR110 KI-VGluT2 mice | | | | |
| --- | --- | --- | --- | --- |
| **Symbol** | **Entrez Gene Name** | **Expr Fold Change** | **Location** | **Type(s)** |
| AKT1^1,2^ | AKT serine/threonine kinase 1 | 1.018 | Cytoplasm | Kinase |
| FARP1^1^ | FERM, ARH/RhoGEF and pleckstrin domain protein 1 | 1.234 | Plasma Membrane | Other |
| GLS^2^ | Glutaminase | 1.046 | Cytoplasm | Enzyme |
| GRIK2^2^ | Glutamate ionotropic receptor kainate type subunit 2 | 1.053 | Plasma Membrane | Ion channel |
| PLCH2^2^ | Phospholipase C eta 2 | 1.122 | Cytoplasm | Enzyme |
| PPP3CB^2^ | Protein phosphatase 3 catalytic subunit beta | 1.024 | Plasma Membrane | Phosphatase |
| WASF1^1^ | WASP family member 1 | 1.149 | Nucleus | Other |
